# Human CD8+ CD57- T_EMRA_ cells: Too young to be called "old"

**DOI:** 10.1371/journal.pone.0177405

**Published:** 2017-05-08

**Authors:** Kriti Verma, Justyna Ogonek, Pavankumar Reddy Varanasi, Susanne Luther, Ivonne Bünting, Katrin Thomay, Yvonne Lisa Behrens, Eva Mischak-Weissinger, Lothar Hambach

**Affiliations:** 1 Dept. of Hematology, Hemostasis, Oncology and Stem Cell Transplantation, Hannover Medical School, Hannover, Germany; 2 Integrated Research and Treatment Center for Transplantation (IFB-Tx), Hannover, Germany; 3 Institute of Human Genetics, Hannover Medical School, Hannover, Germany; Jackson Laboratory, UNITED STATES

## Abstract

End-stage differentiation of antigen-specific T-cells may precede loss of immune responses against e.g. viral infections after allogeneic stem cell transplantation (SCT). Antigen-specific CD8+ T-cells detected by HLA/peptide multimers largely comprise CD45RA-/CCR7- effector memory (T_EM_) and CD45RA+/CCR7- T_EMRA_ subsets. A majority of terminally differentiated T-cells is considered to be part of the heterogeneous T_EMRA_ subset. The senescence marker CD57 has been functionally described in memory T-cells mainly composed of central memory (T_CM_) and T_EM_ cells. However, its role specifically in T_EMRA_ cells remained undefined. Here, we investigated the relevance of CD57 to separate human CD8+ T_EMRA_ cells into functionally distinct subsets. CD57- CD8+ T_EMRA_ cells isolated from healthy donors had considerably longer telomeres and showed significantly more BrdU uptake and IFN-γ release upon stimulation compared to the CD57+ counterpart. Cytomegalovirus (CMV) specific T-cells isolated from patients after allogeneic SCT were purified into CD57+ and CD57- T_EMRA_ subsets. CMV specific CD57- T_EMRA_ cells had longer telomeres and a considerably higher CMV peptide sensitivity in BrdU uptake and IFN-γ release assays compared to CD57+ T_EMRA_ cells. In contrast, CD57+ and CD57- T_EMRA_ cells showed comparable peptide specific cytotoxicity. Finally, CD57- CD8+ T_EMRA_ cells partially changed phenotypically into T_EM_ cells and gained CD57 expression, while CD57+ CD8+ T_EMRA_ cells hardly changed phenotypically and showed considerable cell death after in vitro stimulation. To the best of our knowledge, these data show for the first time that CD57 separates CD8+ T_EMRA_ cells into a terminally differentiated CD57+ population and a so far functionally undescribed “young” CD57- T_EMRA_ subset with high proliferative capacity and differentiation plasticity.

## Introduction

Monitoring of antigen specific CD8+ memory T cells plays an increasing role after allogeneic stem cell transplantation (SCT) in order to evaluate the efficacy and fate of immune responses against e.g. viral infections [1] or transplantation antigens [2]. Particularly, end-stage differentiation of antigen-specific CD8+ T-cells may precede loss of immune responses. CD8+ memory T cells arise from naïve T cells upon antigen encounter [3] and are functionally very heterogeneous. Human CD8+T cells are commonly classified into four subsets based on the surface expression of the leukocyte common antigen isoform CD45RA and the lymph node addressin CCR7 [4]. Thereby, naïve T_N_ cells (CD45RA+/CCR7+) are separated from central memory T_CM_ (CD45RA-/CCR7+), effector memory T_EM_ (CD45RA-/CCR7-) and T_EMRA_ (CD45RA+/CCR7-) T cells [4, 5]. T_CM_ cells show a high proliferative potential, but a poor effector function. Conversely, T_EM_ cells have an immediate effector function but only limited proliferative potential [6]. In man, the developmental relationship among T_CM_, T_EM_ and effector cells is still controversial and has been recently reviewed in detail [7, 8].

Antigen-specific CD8+ T cells detected by HLA/peptide multimer staining largely comprise T_EM_ and T_EMRA_ subsets. However, the relative distribution of T_EM_ and T_EMRA_ may vary considerably depending on the target antigen. For instance, HIV-specific T cells are largely T_EM_ while CMV-specific T cells are mainly of the T_EMRA_ phenotype [9–12]. To date, the experimental evidence on the functional characterization of T_EMRA_ cells is controversial. Several authors consider T_EMRA_ cells overall as the terminally differentiated effector cells supported by low Interleukin-2 and high interferon gamma secretion [4], high cytotoxicity [3], low proliferative capacity and high sensitivity to apoptosis [13]. In contrast, Rufer et al. described heterogeneity within the T_EMRA_ cells and identified CD27+/CD28+/- cells as an intermediate phenotype between naïve and effector cells and CD27-/CD28- cells as late differentiated highly cytotoxic T cells [14]. However, the complexity of subsets with partial functional overlap challenges the longitudinal phenotypical characterization of antigen specific CTLs in the peripheral blood of patients due to their low frequencies and the small available sample sizes. The cell surface molecule CD57, also known as Human Natural Killer 1 (HNK1), might help to reduce the complexity of markers by separating CD8+ T_EMRA_ cells in only two distinct subsets. Brenchley et al. reported that CD57 associates functionally with short telomeres, high sensitivity to apoptosis and replicative senescence in mixed CD8+ memory T cell subsets [15]. Moreover, CD57 expression on CD8+ memory T cells has been shown to strongly correlate with high expression of cytolytic enzymes such as perforin and granzyme A/B [16]. However, due to the differentiation markers used in these studies (i.e. CD45RO or CD45RA combined with CD27 or CD28), the T cell subsets in which CD57 had been functionally described mainly comprised T_CM_ and T_EM_ cells but only partially included CD8+ CD45RA-/CCR7- T_EMRA_ cells. Thus, the role of CD57 specifically in the still ambiguous CD8+ T_EMRA_ population remains undefined. While CD8+ T_EMRA_ cells are mostly considered positive for CD57 [4, 17, 18], some authors also describe a heterogeneous CD57 expression [3] supporting a distinctive role of CD57 also in CD8+ T_EMRA_ cells.

In this study, we investigated the relevance of CD57 to separate human CD8+ T_EMRA_ cells into two subsets with distinct functional and differentiation capacities.

## Materials and methods

### PBMC isolation and patient characteristics

Peripheral blood mononuclear cells (PBMCs) were isolated from peripheral blood collected from six healthy donors (3 donors younger and 3 donors older than 45 years) and from 10 patients who underwent allogeneic SCT at the Hannover Medical School (Germany). Patients were treated according to SCT protocols approved by the Institutional Review Board of the Hannover Medical School. Patients and donors gave written informed consent in accordance with the declaration of Helsinki. Analysis was performed with approval of the Institutional Review Board of the Hannover Medical School (2934–2015). 4 males and 6 female patients who received a transplant of bone marrow (1/10) or peripheral stem cells (9/10) at Hannover Medical School between 2012 and 2015 were included in this study. The median age of the patients was 51 years (range 37–66). Underlying diseases were acute myeloid leukaemia (AML), chronic lymphocytic leukaemia (CLL), Non-Hodgkin’s Leukaemia (NHL), Multiple Myeloma (MM), Aplastic anemia (AA) and Myeloproliferative Neoplasm (MPN). 5/10 patients received graft from matched unrelated donors (MUD), 2/10 from mismatched unrelated donor (MMUD) and 3/10 from HLA identical sibling donor. All patients received Cyclosporin (CsA) along with Mycophenolate mofetil (7/10) or methotrexate (3/10) as GvHD prophylaxis. PBMCs were isolated by ficoll gradient, frozen in liquid nitrogen after supplementation in 80% RPMI-1640, 10% fetal calf serum (FCS, Sigma-Aldrich, Missouri, USA) and 10% dimethyl sulfoxide (DMSO, Sigma-Aldrich).

### Immunophenotyping and analysis

PBMCs thawed and cultured overnight in IMDM (Lonza, Basel, Switzerland), supplemented with 10% human serum (HS, Sigma-Aldrich) were labelled with anti-CD8-Alexa Fluor700 (Clone: RPA-T8, BD Biosciences, New Jersey, USA), anti-CD3-PE-Cy7 (Clone: UCHT1), anti-CD45RA-PerCP Cy5.5 (Clone: HI100), anti-CD27-BV605 (Clone: 0323), anti-CD28-APC(Clone: CD28.2), anti-PD-1-BV421(Clone: EH12.2H7) from Biolegend (San Diego, USA), anti-CCR7-PECF594 (Clone: 150603), anti-CD57-FITC (Clone:NK-1, BD Biosciences) along with AlexaFluor750 labeled live/Dead stain (Life Technologies, Carlsbad, USA). Live/dead and CCR7 staining were performed at 37°C for 15 mins followed by staining with the rest of the antibodies at 4°C for 30 mins. Subsequently, cells were washed, resuspended in PBS and acquired on BD^™^ LSR II (BD Biosciences, San Jose, USA). The phenotypic analysis was performed on FlowJo version 7.6.5 (Treestar, Ashland, USA). For phenotyping of CMV specific T cells, PE labeled tetramer HLA/CMV epitope complex (HLA-A*01:01 CMV pp50: VTEHDTLLY; HLA-A*02:01 pp65-NLVPMVATV; HLAA*24:02 pp65-QYDPVAALF; HLA-B*07: 02 pp65-TPRVTGGGAM; HLA-B*08:01 IE1-ELRRKMMYM; HLA-B*35:01 pp65-IPSINVHHY, MBL International, Woburn, USA) was included in the panel with the above mentioned antibodies.

### Expansion of CMV specific CD8+ T cells

PBMCs of CMV IgG+ patients after allogeneic SCT were stained with AlexaFluor750 labeled live dead stain (Life Technologies) at 37°C for 15 min followed by staining with anti-CD3-AlexaFluor700, anti-CD8-PECy7 and PE-labeled CMV tetramers (Patient 1: HLA-A*01:01/CMV pp50: VTEHDTLLY; Patient 2 and 3: HLA-B*08:01/CMV IE1: ELRRKMMYM) at room temperature for 30 min. Subsequently, 1x10^3^ live CD3/CD8/CMV tetramer+ cells per well were sorted directly into round bottom 96 well plate and cultured in 10% HS/IMDM in the presence of 1% penicillin/streptomycin, Gentamycin (5mg/ml, Life Technologies) Fungisone (0.5mg/ml, Life Technologies), 1x10^5^ autologous PBMCs irradiated at 30Gy, 1% Leucoagglutinin PHA-L (1 μg/mL, Sigma-Aldrich) and supplementation of 120 IU/ml interleukin-2 (IL-2, ImmunoTools, Friesoythe, Germany) every 2–3 days for 2–3 weeks. Re-stimulation was performed with 1% LeucoA and irradiated autologous feeder cells every 7–10 days. CMV CTL lines were frozen after 3 weeks in culture.

### Sorting of CD8+ T cell subsets

After thawing and culturing overnight in 10% HS / IMDM, PBMCs were labelled with anti-CD8-Alexa Fluor700, anti-CD3-PE-Cy7, anti-CD45RA-PerCP Cy5.5, anti-CCR7-PECF594, anti-CD57-FITC, anti-PD-1-BV421, anti-CD27-BV605, anti-CD28-APC and AlexaFluor750 labeled live/Dead stain. Live/dead and CCR7 staining were performed at 37°C for 15 mins followed by staining with the rest of the antibodies at 4°C for 30 mins. Subsequently, cells were sorted on FACS Aria^™^ II (BD Biosciences). CD57- and CD57+ T_EMRA_ populations of in vitro expanded CMV tetramer+ T cells were separated on FACS Aria^™^ II (BD Biosciences) after resting in 10% HS/IMDM supplemented with 120 IU/ml of IL-2 for three days and staining with live/Dead stain, anti-CD45RA-PerCP Cy5.5 (Biolegend), CMV Tetramer-PE (MBL International), anti-CD57-FITC and anti-CD4-BV421 at RT for 30 mins. Post-sorting analysis of purified subsets revealed greater than 98% purity. Subsequently, sorted T cell subsets were directly subjected to functional assays.

### Cell proliferation assay

The proliferative capacity of T cell subsets of healthy donors and of CMV CTLs was measured by quantification of 5-bromo-2’deoxyuridine (BrdU) incorporation. 1 × 10^4^ T cells/well were sorted directly into flat bottom 96-well microtiter plates. T cell subsets of healthy donors were stimulated with 2% Leucoagglutinin PHA-L (1 μg/mL, Sigma-Aldrich) in a final volume of 0.2 mL/well in the presence of 2× 10^4^ autologous PBMCs irradiated at 100Gy. CMV CTL subsets were stimulated with CD14+ MACS isolated monocytes with the relevant peptides (Patient 1: HLA-A*01:01/CMV pp50: VTEHDTLLY; Patient 2 and 3: HLA-B*08:01/IE1-ELRRKMMYM) at different concentrations added directly to the well. After 3 days, BrdU was added. On day 4 supernatant was collected for subsequent assays and incorporated BrdU was quantified by enzyme-linked immunosorbent assay (ELISA) according to the manufacturer’s protocol (Roche, Basel, Switzerland). The ELISA plate was read at 370 nm (reference 492 nm) in an ELISA reader. The collected supernatant was stored at -20°C for measuring secreted Interferon **γ** (IFN-γ) levels.

### Interferon-γ release

IFN-γ levels in cell culture supernatants collected from BrdU uptake assay was measured by ELISA kit (eBioscience, Vienna, Austria) according to the manufacturer’s instructions. The ELISA plate was read at 450 nm (reference 570 nm) in an ELISA reader.

### Cytotoxicity assay

PHA blasts (PHAb) positive for the relevant HLA were generated by stimulating PBMCs with 1% Leucoagglutinin PHA-L along with 120IU/IL-2 supplementation every 2–3 days for two weeks in culture. CD4+ PHA blasts (PHAb) were further enriched by MACS and frozen for subsequent use as target cells for CD8 + T cell mediated peptide specific lysis. CD4+ PHAb were thawed and cultured overnight at 37°C in the presence of 120 IU/ml IL-2, labeled with 3 μM CFSE (Life Technology) in 1 ml 10% HS/IMDM for 10 minutes at 37°C. The reaction was stopped by 2 ml 10% HS/IMDM, followed by 2 min incubation at 4°C. After washing twice in PBS, 5 × 10^3^ CFSE labeled PHAb in 50 μL 10% HS/IMDM per well were added to a V-bottom 96-well microtiter plate. The relevant peptide concentration in 50 μL 10% HS/IMDM was added and incubated at 37°C for 60 min. Finally, 1 × 10^4^ CMV CTLs were added in a total volume of 150 μL 10% HS/IMDM per well and the plate was centrifuged at 1500 rpm for 5 min without break and incubated further for 4h at 37°C. Subsequently, cells were stained for anti-CD4-BV421 (Clone: OKT4, Biolegend) as control for the exclusion of intercellular CFSE transfer and anti-CD8-PECy7 (Clone: RPA-T8, BD Biosciences) to identify the effector cells at 4°C for 30 min. Wells were harvested, 40,000 Flow-Count Fluorospheres and 7AAD (Beckman Coulter) were added just prior to acquisition on BD LSR II. 5000 microbeads were acquired for each sample. Specific lysis of target cells was calculated as: % specific lysis = % dead target cells with effector cells—% dead target cells without effector cells, as previously published [19].

### Absolute telomere length measurement

Absolute telomere length was measured by real time PCR as previously published [20] with slight modifications including a pre-amplification step for application on small cell numbers [21]. 50 cells were sorted in triplicate directly into 4 μl lysis buffer per well of a V-bottom 96 well plate and frozen for subsequent PCR at -20°C. The pre-amplification was performed on the lysate using the telomere primers (forward: 5′ (TTAGGG)14 3′ and reverse:5′ CAGCAAGTGGGAAGGTGTAATCCGTCTCCACAGACAAGGCCAGGACTCGTTTG 3′) and the single copy reference gene 36B4 primers (forward: 5′ CAGCAAGTGGGAAGGTGTAATCC-3′ and reverse: 5′ CAGCAAGTGGGAAGGTGTAATCCGTCTCCACAGACAAGGCCAGGACTCGTTTG 3′) with the reaction conditions as described [20]. The pre-amplification product was purified using the Zymo PCR clean and concentration kit (Zymo Research, CA, USA). The final elution was made in 44 μl elution buffer. Purified PCR product was used in the subsequent real time PCR using the same primers as above and reaction conditions as described [20]. The methodology was validated for established tumor cell lines of known telomere length and T cell clones by Southern Blot analysis at the Department of Human Genetics, MHH, Hannover (S1 Fig).

### Statistics

All statistical analysis was performed using Prism 5 (GraphPad, California, USA). A p value < 0.05 was considered statistically significant.

## Results

### Distribution of CD57 in CD8+ T cell subsets

**Fig 1:**
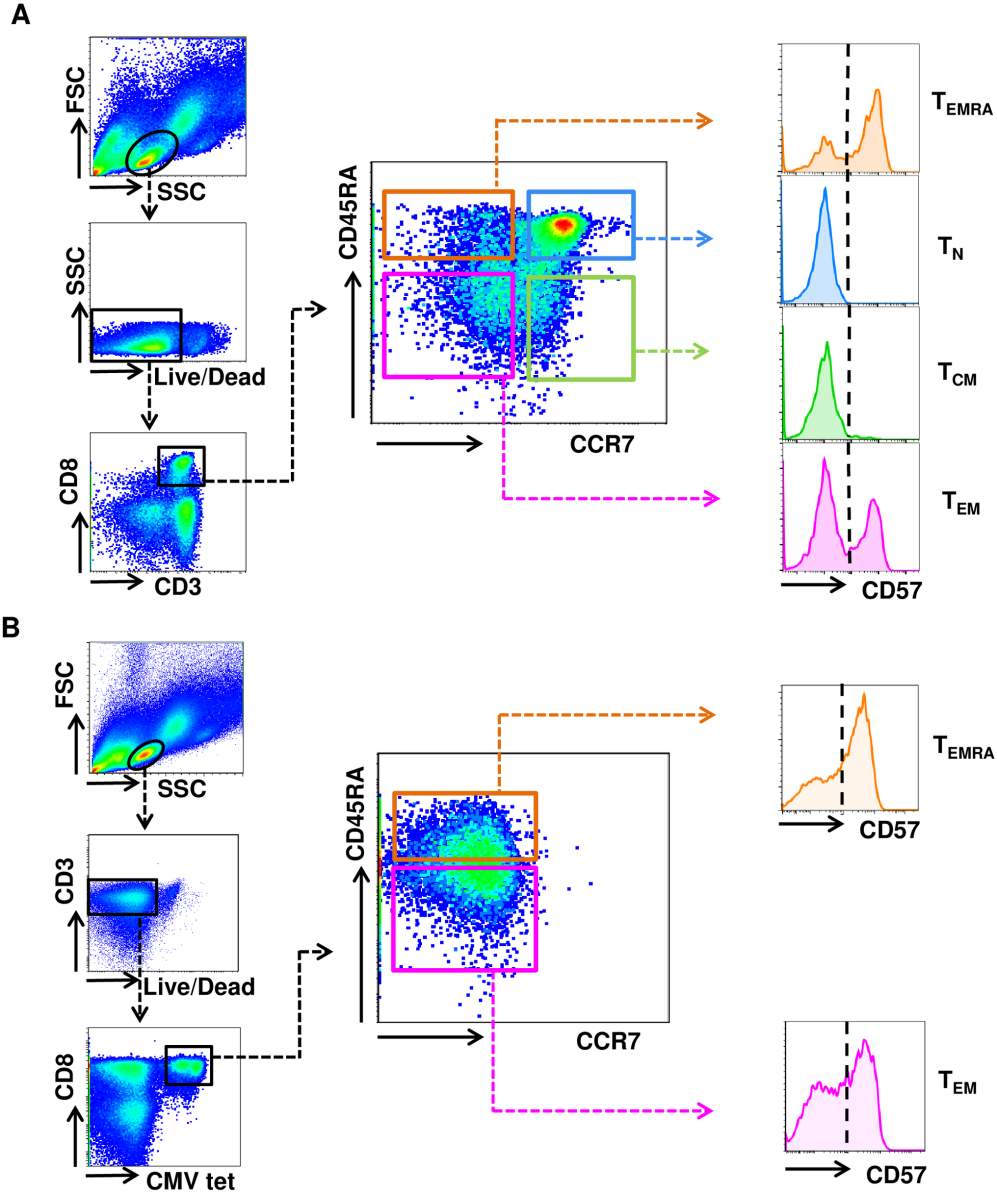
Distribution of CD57+ cells in CD8+ T cell subsets. (A-B) Gating strategy for the assessment of the CD57 distribution in subsets of (A) overall CD8+ T cells and (B) CMV specific CD8+ T cells. (A) Shown is one representative example of the CD57 distribution within CD8+ T_N_, T_CM_, T_EM_ and T_EMRA_ cells of 6 healthy individuals. (B) Shown is one representative example of the CD57 distribution within CD8+ CMV tetramer+ T_EM_ and T_EMRA_ cells of 10 patients after allogeneic SCT.

Firstly, the distribution of CD57 in CD8+ T cell subsets in the peripheral blood of six healthy donors was analyzed using 10-color flow cytometry. A representative example of this analysis is shown in Fig 1A. The CD8+ T cells comprised 40% (+/- 21) T_N_ cells, 13% (+/- 18), T_CM_ cells, 18% (+/- 5) T_EM_ and 11% (+/- 6) T_EMRA_ cells. All T_N_ and T_CM_ cells were CD57-, whereas T_EM_ and T_EMRA_ cells segregated into 32% (+/- 18) and 41% (+/- 12) CD57+ cells, respectively (Table 1). To investigate the CD57 distribution in antigen specific T cells, further studies were applied on CMV specific CD8+ T cells emerging after allogeneic SCT due to the clinical relevance of CMV infections in transplantation and the relative abundance of CMV specific T cells technically facilitating functional experiments. HLA/CMV peptide tetramers were used to identify CMV specific CD8+ T cells in the peripheral blood of 10 patients collected at median 139 days (min. 38/ max. 354) after allogeneic HLA-matched SCT. In accordance with previous reports, there were overall more T_EM_ and T_EMRA_ cells compared to healthy donors [9, 10]. CMV tetramer+ CD8+ T cells comprised only of T_EM_ and T_EMRA_ cells with 43% (+/-21) and 56% (+/-21), respectively (Table 1). Further investigation revealed that CD8+ CMV tetramer+ T_EM_ and T_EMRA_ cells segregated into 69% (+/- 21) and 58% (+/- 25) CD57+ cells, respectively (Table 1). A representative example of this analysis is shown in Fig 1B.

**Table 1:** Distribution of subsets in CD8+ T cells of healthy donors and CMV specific CTLs. Mean percentage distribution (± standard deviation) of CD8+ T cell subsets and CD57+/- cells in CD8+ T cell subsets is depicted.

	Healthy donors (n = 6)			Transplanted patients (n = 10)					
				CMV Tetramer -			CMV Tetramer +		
Subsets	Total %	CD57- %	CD57+ %	Total %	CD57- %	CD57+ %	Total %	CD57- %	CD57+ %
**T**_**N**_	**40(±21)**	**100(±0)**	**0(±0)**	**8(±5)**	**100**	**0**	**0**	**0**	**0**
**T**_**CM**_	**13(±18)**	**100(±1)**	**0(±1)**	**12(±10)**	**100**	**0**	**0**	**0**	**0**
**T**_**EM**_	**18(±5)**	**68(±18)**	**32(±18)**	**31(±18)**	**41(±19)**	**59(±19)**	**43(±21)**	**31(±21)**	**69(±21)**
**T**_**EMRA**_	**11(±6)**	**59(±12)**	**41(±12)**	**48(±23)**	**55(±12)**	**45(±12)**	**56(±21)**	**42(±25)**	**58(±25)**

### Functional characterization of CD57+/- CD8+ T_EM_ and T_EMRA_ subsets

**Fig 2:**
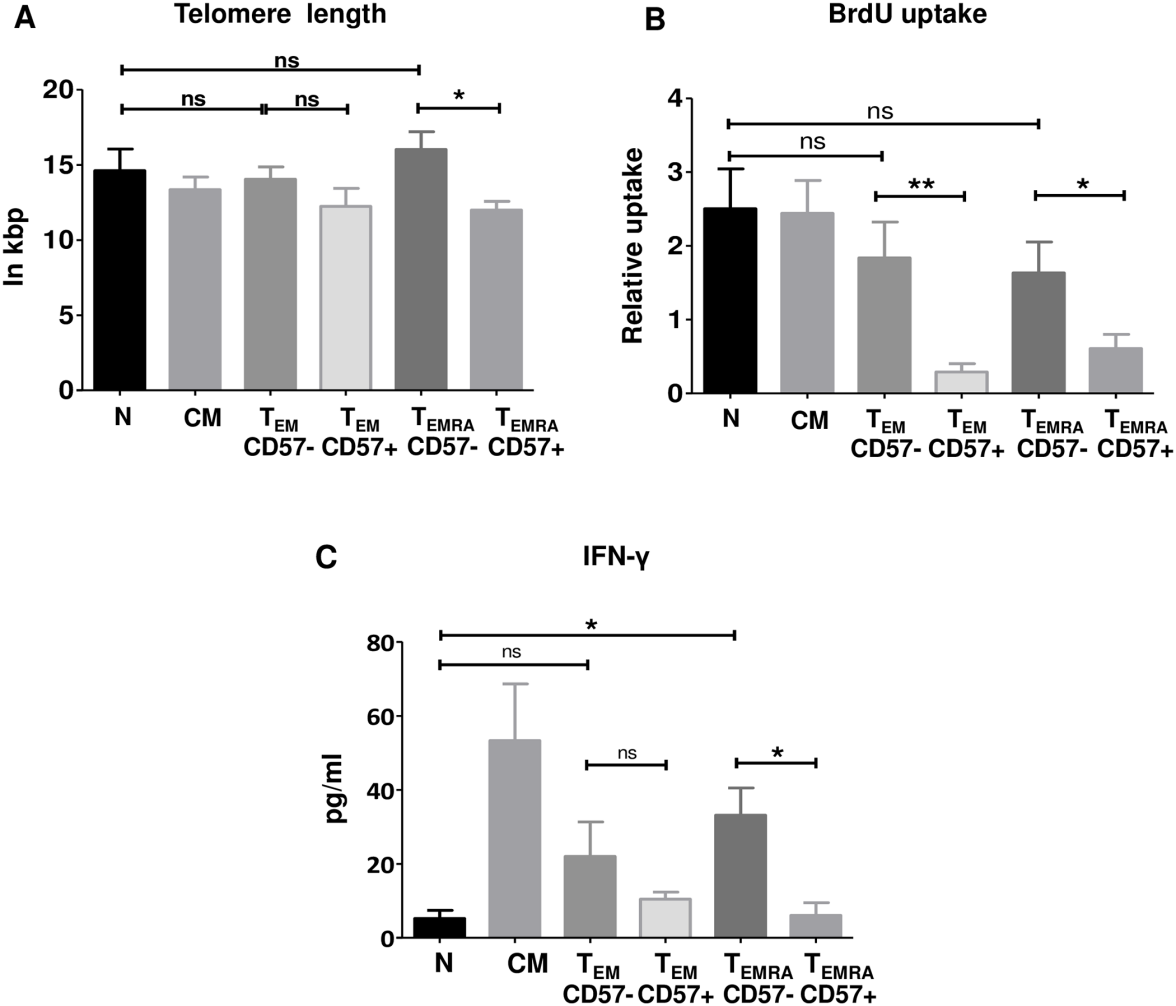
Functional characterization CD8+ T cell subsets from healthy donors. (A-C) CD8+ T_N_, T_CM_, T_EM_ and T_EMRA_ cells were highly purified by FACS sorting from the peripheral blood of healthy donors according to the cell surface markers CD45RA, CCR7 and further subdivided by CD57. Subsequently, sorted T cells were stimulated with PHA and IL-2 supplementation every two days. Shown are the results from 5 independent healthy donors. (A) Absolute telomere length directly after sorting. (B) BrdU uptake 5 days after stimulation. (C) IFN-γ released in the supernatant 5 days after stimulation. Significance was calculated using Mann Whitney test. * indicates p<0.05, ** indicates p<0.01, ns indicates not significant. Only statistical comparisons between T_N_ and CD57+/- T_EM_ / T_EMRA_ cells and among CD57+/- T_EM_ / T_EMRA_ cells are shown.

In order to investigate the functional differences between CD8+ T_N_, T_CM_, CD57+/- T_EM_ and CD57+/- T_EMRA_ subsets, CD8+ T cells were enriched from the peripheral blood of healthy donors and subsequently FACS sorted into T_N_, T_CM_, CD57+ and CD57- T_EM_ and T_EMRA_ cells. The sorted cells were analyzed for telomere length and—subsequent to PHA and IL-2 stimulation—for BrdU uptake and IFN-γ release. Longest and comparable telomeres were found for T_N_, CD57- T_EM_ and CD57- T_EMRA_ cells (Fig 2A). CD57+ T_EM_ cells had shorter telomeres than CD57- T_EM_ cells by trend. Additionally, telomere length was significantly shorter in CD57+ compared to CD57- T_EMRA_ cells (p = 0.016, Mann-Whitney U test, Fig 2A). There were no significant differences in BrdU uptake between T_N_ and T_CM_, CD57- T_EM_ and CD57- T_EMRA_ cells (Fig 2B). Conversely, CD57+ cells showed a significantly lower BrdU uptake compared to CD57- cells both in T_EM_ and T_EMRA_ subsets (p = 0.004 and p = 0.026, respectively; Mann-Whitney U test; Fig 2B). IFN-γ release was found to be highest for T_CM_ and low for T_N_, CD57+ T_EM_ and T_EMRA_ cells. CD57+ cells released less IFN-γ compared to CD57- cells both in T_EM_ and T_EMRA_ subsets. However, this difference between CD57- and CD57+ cells was significant only for T_EMRA_ cells (p = 0.032; Mann-Whitney U test; Fig 2C).

### Functional characterization of CMV specific CD57+/- CD8+ T_EMRA_ subsets

**Fig 3:**
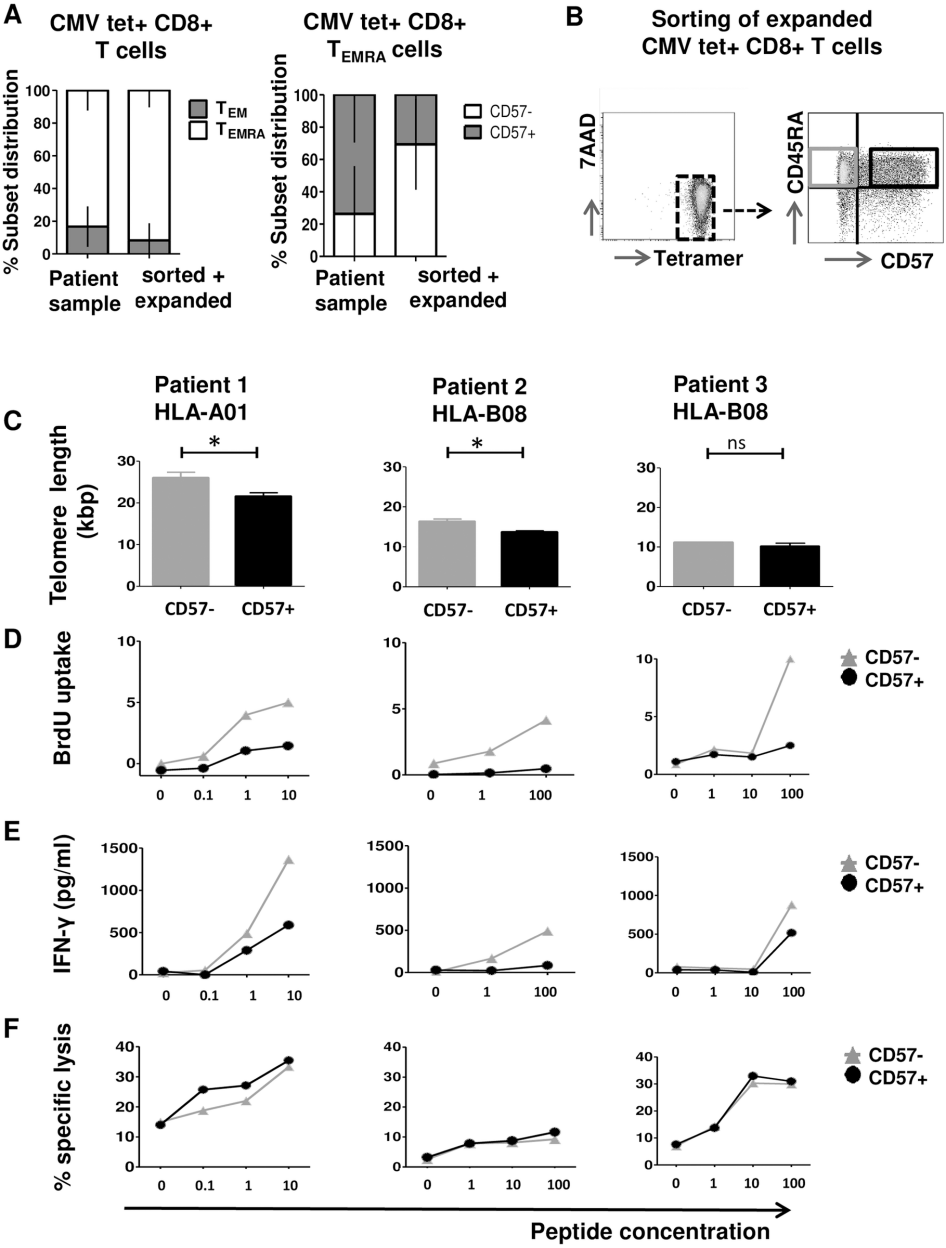
Phenotypic and functional characterization of CMV tetramer+ cells. **(A)** CD8+ CMV tetramer+ T cells were FACS sorted from the peripheral blood of 3 patients after allogeneic SCT and in vitro expanded on autologous feeder cells. Depicted is the T_EM_ and T_EMRA_ subset distribution within CD8+ CMV HLA/tetramer+ T cells (left) and CD57+ distribution within CD8+ CMV HLA/tetramer+ T_EMRA_ cells (right) in the peripheral blood compared to after in vitro expansion of FACS sorted CD8+ CMV tetramer+ T cells. Y-axis: % subset distribution within CD8+ CMV HLA/tetramer+ T cells and CD8+ CMV HLA/tetramer+ T_EMRA_ cells. Error bars indicate standard deviation. (B) Sorting strategy for viable in vitro expanded CD8+ CMV HLA/tetramer+ CD8+ T cells for CD45RA and CD57 allowing functional analysis. (C) Absolute telomere length directly after sorting. (D) BrdU uptake 4 days after stimulation with CD14+ monocytes loaded with increasing concentrations of the relevant HLA/CMV peptide. (E) INF-γ release in the supernatant from the BrdU uptake assay. (F) Specific lysis of CFSE labelled PHA blasts loaded with increasing concentrations of the relevant HLA/CMV peptide. Significance was calculated using Mann-Whitney-U test. * indicates p<0.05, ** indicates p<0.01, ns indicates not significant.

In order to get further insights into the relevance of CD57 in CD8+ antigen specific T_EMRA_ cells, the functional properties of CD8+ CMV specific T_EMRA_ subsets with and without CD57 expression were compared. CMV tetramer+ CD8+T cells were isolated from the peripheral blood of 3 patients after allogeneic SCT by FACS sorting and expanded on autologous feeder cells in vitro to cell numbers sufficient for functional studies. Phenotypic analysis of CMV tetramer+ CD8+T cells for CD45RA/CCR7 in vivo and after sorting and expansion in vitro revealed a comparable distribution of T_EM_ and T_EMRA_ subsets with a trend towards more CD57- cells within the T_EMRA_ subset (Fig 3A). Subsequently, these in vitro expanded CMV specific CTLs were further purified into CD57+ and CD57- T_EMRA_ subsets by FACS (Fig 3B). The CD57+ and CD57- T_EMRA_ cells were directly analyzed for telomere length (Fig 3C) and for CMV peptide dependent BrdU uptake (Fig 3D), IFN-γ release (Fig 3E) and cytotoxicity (Fig 3F). CD57+ T_EMRA_ cells showed significantly shorter telomeres compared to CD57- T_EMRA_ cells in 2/3 patients derived CMV CD8+ T cells (Fig 3C). BrdU uptake and IFN-γ release upon stimulation with target cells loaded with increasing CMV peptide concentrations revealed considerably higher BrdU uptake (Fig 3D) and IFN-γ release (Fig 3E) for CD57- T_EMRA_ cells than for CD57+ T_EMRA_ cells. In contrast, CD57+ T_EMRA_ cells showed only slightly higher cytotoxicity against targets loaded with increasing concentrations of CMV peptide than CD57- T_EMRA_ cells (Fig 3F).

### Differentiation potential of CD8+ CD57- and CD57+ T_EMRA_ cells

**Fig 4:**
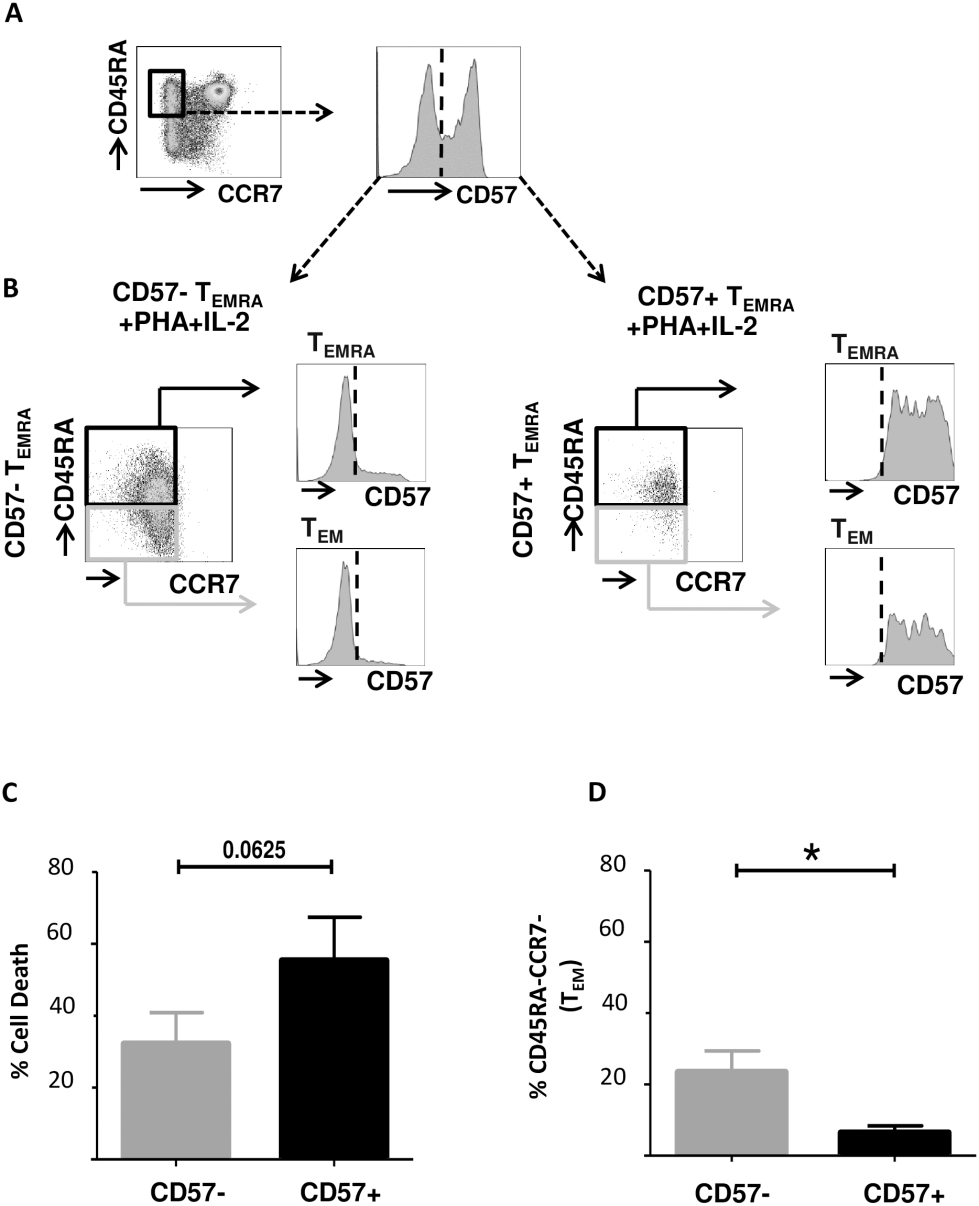
Differentiation potential of CD8+ CD57- and CD57+ T_EMRA_ cells. (A) CD8+ T_EMRA_ cells were highly purified by FACS sorting from the peripheral blood of 5 healthy donors according to the cell surface markers CD45RA, CCR7 and further subdivided by CD57. Representative example of the sorting strategy is shown. (B) Subsequently, sorted T cells were stimulated with PHA and 120IU/ml IL-2 supplementation every two days. Phenotypic analysis of CD57- and CD57+ T_EMRA_ after 7 days in culture is shown in a representative example. (C) Percentage of cell death in sorted CD57- and CD57+ T_EMRA_ cells after 7 days in culture upon PHA+ IL-2 stimulation. (D) Percentage of sorted CD57- and CD57+ T_EMRA_ cells that lose expression of CD45RA after 7 days in culture upon PHA+ IL-2 stimulation. Significance was calculated using Wilcoxon matched-pairs signed rank test, * indicates p<0.05.

Finally, the fate of CD8+ CD57+ and CD57- T_EMRA_ cells after PHA +IL-2 stimulation was studied in in vitro. CD8+ CD57+ and CD57- T_EMRA_ cells were highly purified from peripheral blood of 5 healthy donors by FACS sorting (Fig 4A). The purity was confirmed by FACS analysis to be >98% after sorting. Subsequently, sorted T cell subsets were stimulated with PHA supplemented with 120 IU/ml IL-2 every two days and kept in culture for 7 days. Subsequently, T cells were phenotypically characterized based on cell surface expression of CD45RA, CCR7 and CD57 (Fig 4B). Overall CD57- T_EMRA_ cells by trend were less susceptible to cell death upon PHA + IL-2 stimulation than CD57+ T_EMRA_ cells (p = 0.0625, Wilcoxon matched-pairs signed rank test, Fig 4C). There was a significant loss of CD45RA surface expression on CD57- T_EMRA_ cells compared to CD57+ T_EMRA_ cells (p = 0.0355, Wilcoxon matched-pairs signed rank test, Fig 4D). Moreover, 12(+/-8) % of CD57- T_EMRA_ cells acquired CD57 expression (exemplified in Fig 4B). These findings, suggest that CD57- T_EMRA_ cells show differentiation potential towards T_EM_, which is largely absent on CD57+ T_EMRA_ cells.

## Discussion

In this study, we have shown for the first time experimentally the relevance of CD57 as a marker to separate human antigen specific CD8+ T_EMRA_ cells into functionally distinct subsets. Thereby, we functionally characterized a previously undescribed “young” CD57- T_EMRA_ population that differs by its high proliferative and differentiation plasticity from its CD57+ terminally differentiated counterpart. So far, phenotypic characterization of human antigen specific CD8+ T cells is commonly based on the CD45RA/CCR7 marker system. Among the memory subsets, the CD8+ CD45RA+CCR7- T_EMRA_ cells had been either interpreted as terminally differentiated T cells in total [4] or they were often excluded from interpretation in monitoring studies of antigen specific immune responses due to the complexity of subdivision using current phenotypic markers [22, 23]. Despite CD57 is mostly considered as general T cell senescence marker [15] its role specifically in CD8+ T_EMRA_ cells was so far undefined. Our data show that around 11% of CD8+ T cells in the peripheral blood of healthy donors are T_EMRA_ cells, of which 41% are CD57+. These data are comparable to previous data of Hamann et al. describing around 50% of T_EMRA_ cells being CD57+ and support the previously described phenotypical heterogeneity of CD8+ T_EMRA_ cells [3, 14]. Moreover, 56% of CD8+ CMV specific CTLs after allogeneic SCT are T_EMRA_ cells, of which 58% are CD57+. These data are comparable to previous findings showing that CMV-specific T cells are mainly of the T_EMRA_ phenotype [9–12]. Healthy donor derived CD8+ CD57- T_EMRA_ cells showed a telomere length that was by trend even longer than that of T_N_ cells, while telomere lengths of CD57+ T_EMRA_ cells were the shortest measured in our tested T cell subsets. Also previous reports showed that CD8+ T_EMRA_ cells can be subdivided into populations with long [14] and short [24] telomeres, however based on the differentiation marker system CD27 and CD28. The long telomeres in CD57- T_EMRA_ cells associated well with high T cell proliferation and INF-γ release in our study, while short telomeres in CD57+ T_EMRA_ cells associated with a low proliferative response and INF-γ release in response to PHA stimulation. Similarly, highly purified CMV specific CD57- T_EMRA_ cells showed a considerably higher sensitivity in response to CMV peptides with regard to T cell proliferation and INF-γ release but slightly lower peptide sensitivity in cytotoxicity assays compared to CMV specific CD57+ T_EMRA_ cells. These data demonstrate that CD57 separates CD8+ T_EMRA_ cells based on a considerably different proliferative capacity. We assumed that this higher proliferative capacity of CD8+ CD57- T_EMRA_ cells might also associate with a higher differentiation capacity compared to the CD57+ counterpart. Our data showed that in vitro stimulation of highly purified CD8+ CD57- T_EMRA_ cells resulted in a partial loss of CD45RA suggesting the emergence of T_EM_ cells. Additionally, some CD8+ CD57- T_EMRA_ also gained CD57 expression. In contrast, CD8+ CD57+ T_EMRA_ cells hardly changed phenotypically and were more susceptible to cell death than CD8+ CD57- T_EMRA_ cells after stimulation. These data indicate that the CD57- T_EMRA_ cells exhibit differentiation plasticity absent in CD57+ subset. Evidently, these observations do not elucidate the still enigmatic precursors of T_EMRA_ cells. Rufer et al. suggested, based on measurement of T-cell receptor excision circles (TRECs) which indicate maturation of T cells, that the CD8+ CD27+ T_EMRA_ subset comprises cells that are evolving from a naïve differentiation stage [14]. Potentially, the position of CD57- T_EMRA_ cells within the differentiation pathway of human T cells needs to be refined based on studies on whether CD57- T_EMRA_ cells might even directly arise from T_N_ cells.

The capacity of CD57 to separate CD8+ T_EMRA_ cells into subsets with contrasting functional and developmental properties may have a considerable impact on the monitoring of antigen specific CTLs in patients after HSCT. Evidently, longitudinal studies on immune responses are often limited by the low frequency of antigen specific T cells in the peripheral blood and by the available patient sample size [25]. Therefore, phenotypic markers of polychromatic panels for the characterization of antigen specific CTLs need to be limited to a minimum in order to end up with subpopulation sizes large enough to allow statistical comparisons. Consequently, the selected markers should not provide an informative overlap [18]. Phenotypic analysis of the CD8+ T_EMRA_ population in healthy donors revealed that only 63 (+/-9) % of CD27-/CD28- T_EMRA_ cells are positive for CD57 (S1 Table). Conversely, 95 (+/-2)% of CD57+ T_EMRA_ cells are CD27-/CD28- (S2 Table) which were shown to have end stage differentiation properties [14]. These data suggest that CD27-/CD28- T_EMRA_ cells are not a homogenous population based on CD57 expression and that CD57+ T_EMRA_ cells are a subset of CD27-/CD28- T_EMRA_ cells. Since 37 (+/- 9)% of CD27-/CD28- cells are also CD57- (S1 Table) and absence of CD57 indicated high proliferative potential in our and other studies [16], CD27-/CD28- cells are not entirely terminally differentiated. Thus, in contrast to absence of both CD27 and CD28, CD57 might be an excellent marker to uniquely distinguish terminally differentiated CD8+ T_EMRA_ from others. Thereby, CD57 may help to restrict the number of functionally relevant markers necessary to characterize T_EMRA_ cells in studies monitoring antigen specific T cell responses. The clinical importance of CD57 as a singular marker had been previously shown by Scheinberg et al. who found that negativity for CD57 predicts long-term persistence of donor derived CD45RO+ CD27- CMV specific T cells in the recipient and confers protection against viral reactivation after HSCT [26]. Additional studies are required to assess whether also the absence of CD57 expression on CMV specific T_EMRA_ cells (which are CD45RO-) associates with the persistence of CMV immune responses after allogeneic SCT.

In conclusion, CD57 alone might reduce the complexity of currently used phenotypic markers in polychromatic panels to identify end-stage differentiated CD8+ T_EMRA_ cells. Transcriptional profiling of the CD57+/- T_EMRA_ cells may further help in confirming the functional role of CD57 and in defining the differentiation status of T_EMRA_ cells. Finally, we have shown that CD57 separates CD8+ T_EMRA_ cells into a terminally differentiated CD57+ population and a so far functionally undescribed “young” CD57- T_EMRA_ subset with high proliferative capacity and differentiation plasticity.

## Supporting information

S1 Fig Validation of absolute telomere length quantification. Absolute telomere length was quantified by qPCR on DNA isolated from 50 cells including a pre-amplification step and validated by southern blot hybridization using 1μg genomic DNA isolated from 2x106 cells. The range of measurement was defined by absolute telomere length analysis for the human T cell leukaemia cell line 1301 as reference for long telomeres and the breast cancer cell line cal51 as reference for short telomeres. Additionally, a CMV CTL clone of unknown telomere length was measured.
(TIF)

S1 Table Distribution of CD57 within CD8+ CD27+/- CD28+/- T_EMRA_ cell subsets of six healthy donors. Mean percentage (± standard deviation) is depicted for all subsets.
(DOCX)

S2 Table Distribution of CD27 and CD28 within CD57+ and CD57- T_EMRA_ cell subsets of six healthy donors. Mean percentage (± standard deviation) is depicted for all subsets.
(DOCX)
