# Supplementary figures and images for: Human CD8+ CD57- TEMRA cells: Too young to be called "old"
Source: PLoS One. 2017 May 8;12(5):e0177405. doi: 10.1371/journal.pone.0177405 (PMC5421808; doi:10.1371/journal.pone.0177405)

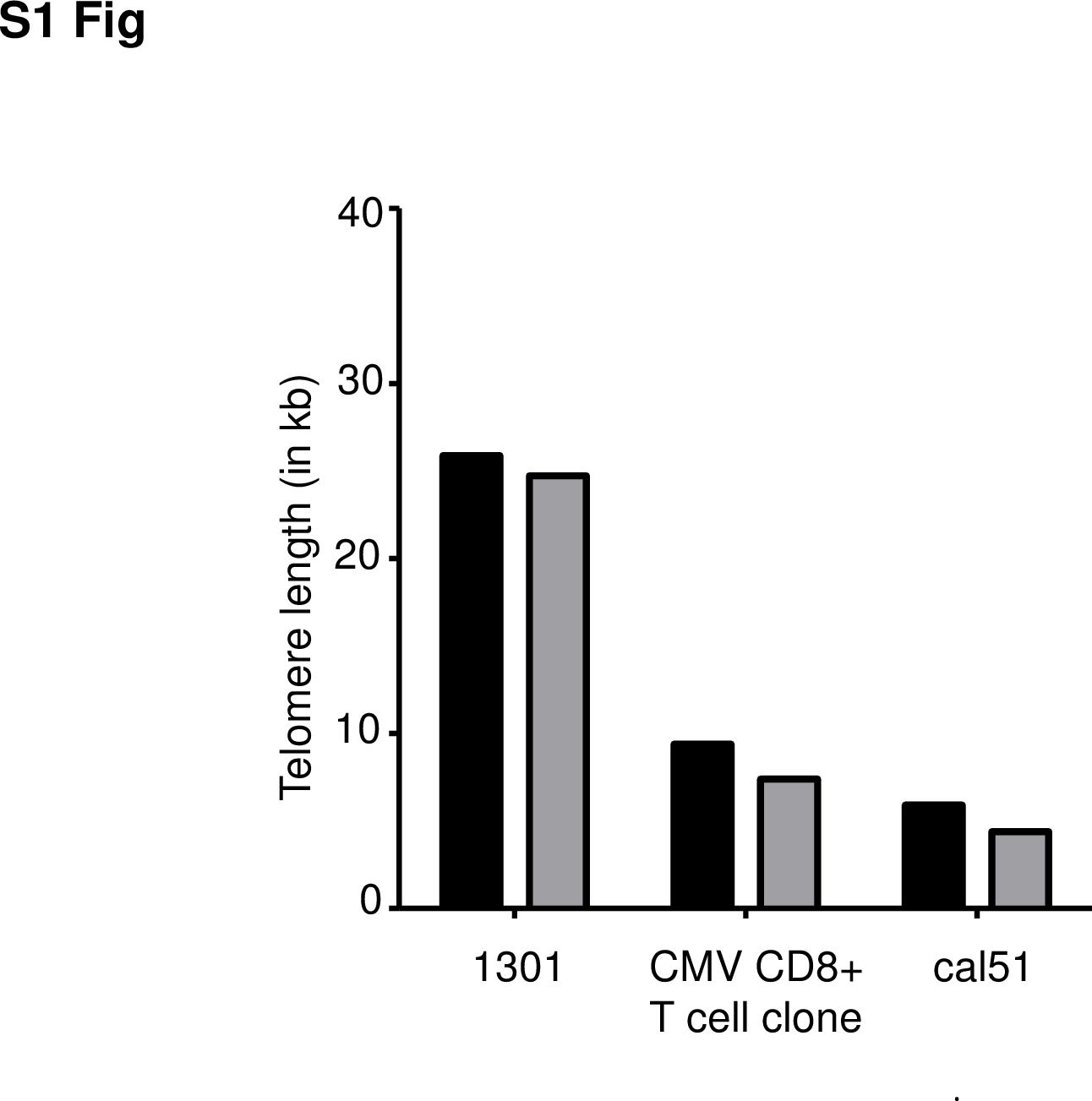

Supplement: S1 Fig — Absolute telomere length was quantified by qPCR on DNA isolated from 50 cells including a pre-amplification step and validated by southern blot hybridization using 1μg genomic DNA isolated from 2x10^6^ cells. The range of measurement was defined by absolute telomere length analysis for the human T cell leukaemia cell line 1301 as reference for long telomeres and the breast cancer cell line cal51 as reference for short telomeres. Additionally, a CMV CTL clone of unknown telomere length was measured. (TIF) [file pone.0177405.s001.tif]
