# Supplementary material for: Human CD8+ CD57- TEMRA cells: Too young to be called "old"
Source: PLoS One. 2017 May 8;12(5):e0177405. doi: 10.1371/journal.pone.0177405 (PMC5421808; doi:10.1371/journal.pone.0177405)
Supplement: S1 Table — Mean percentage (± standard deviation) is depicted for all subsets. (DOCX) [file pone.0177405.s002.docx]

**Supporting Information**

**S1 Table.**

|  | **Subset** | **Total** | **CD57-** | **CD57+** |
| --- | --- | --- | --- | --- |
| **T_EMRA_** | **CD27+/CD28+** | 25(±11) | 99(±1) | 1(±1) |
| **T_EMRA_** | **CD27-/CD28+** | 3(±2) | 96(±9) | 4(±9) |
| **T_EMRA_** | **CD27+/CD28-** | 27(±7) | 73(±16) | 26(±16) |
| **T_EMRA_** | **CD27-/CD28-** | 45(±12) | 37(±9) | 63(±9) |
