# Supplementary material for: Human CD8+ CD57- TEMRA cells: Too young to be called "old"
Source: PLoS One. 2017 May 8;12(5):e0177405. doi: 10.1371/journal.pone.0177405 (PMC5421808; doi:10.1371/journal.pone.0177405)
Supplement: S2 Table — Mean percentage (± standard deviation) is depicted for all subsets. (DOCX) [file pone.0177405.s003.docx]

**Supporting Information**

**S2 Table.**

|  | **Subset** | **Total** | **CD27+/CD28+** | **CD27+/CD28-** | **CD27-/CD28+** | **CD27-/CD28-** |
| --- | --- | --- | --- | --- | --- | --- |
| **T_EMRA_** | **CD57-** | 59 | 36(±15) | 9(±4) | 15(±7) | 41(±13) |
| **T_EMRA_** | **CD57+** | 41 | 1(±1) | 2(±1) | 3(±1) | 95(±2) |
